# QTLRel: an R Package for Genome-wide Association Studies in which Relatedness is a Concern

**DOI:** 10.1186/1471-2156-12-66

**Published:** 2011-07-27

**Authors:** Riyan Cheng, Mark Abney, Abraham A Palmer, Andrew D Skol

**Affiliations:** 1 Department of Human Genetics, The University of Chicago, IL 60637 USA; 2 Department of Psychiatry and Behavioral Neuroscience, The University of Chicago, IL 60637 USA; 3 Department of Medicine, The University of Chicago, IL 60637 USA

## Abstract

**Background:**

Existing software for quantitative trait mapping is either not able to model polygenic variation or does not allow incorporation of more than one genetic variance component. Improperly modeling the genetic relatedness among subjects can result in excessive false positives. We have developed an R package, QTLRel, to enable more flexible modeling of genetic relatedness as well as covariates and non-genetic variance components.

**Results:**

We have successfully used the package to analyze many datasets, including F_34 _body weight data that contains 688 individuals genotyped at 3105 SNP markers and identified 11 QTL. It took 295 seconds to estimate variance components and 70 seconds to perform the genome scan on an Linux machine equipped with a 2.40GHz Intel(R) Core(TM)2 Quad CPU.

**Conclusions:**

QTLRel provides a toolkit for genome-wide association studies that is capable of calculating genetic incidence matrices from pedigrees, estimating variance components, performing genome scans, incorporating interactive covariates and genetic and non-genetic variance components, as well as other functionalities such as multiple-QTL mapping and genome-wide epistasis.

## Background

Methods to search for quantitative trait loci (QTL) in common experimental designs are well established, and software to analyze these populations is widely available. One popular package, R/qtl [1], provides a comprehensive toolset for QTL mapping. Since it does not allow random effects, R/qtl is most suitable for mapping populations such as F_2 _and backcross where individuals are equally genetically related. Software that can model polygenic effects due to genetic relatedness includes TASSEL [2] and EMMA [3]. Both allow covariates as fixed effects but are only capable of incorporating a random term to account for one genetic variance component. However, both additive and dominance modes of inheritance are common for many quantitative traits. Ignoring these variance components may result in excessive false positives. Moreover, researchers may also be interested in interactive covariates, epistasis and non-genetic random effects. We have developed an R package QTLRel that meets all these needs.

## Implementation

### Statistical model

Consider the following statistical model(1)

where ***y ***is a vector of phenotypes, ***β ***is a vector of covariate effects, ***γ ***is a vector of putative QTL effects, ***u ***is a vector of polygenic effects and ***ε ***is a vector of residual effects. ***X***, ***Q ***and ***Z ***are incidence matrices. ***β ***can be fixed, random or a mix of both fixed and random. Assume that ***u ***~ *N*(**0**, ***G***), ***ε ***~ *N*(**0**, ***I**σ*^2^), and ***u ***is independent of ***ε***. ***G ***consists of five genetic variance components including additive and dominance components as well as three other components that model excess similarity due to inbreeding [4]. The incidence matrices corresponding to these five genetic variance components can be obtained from condensed identity coefficients as defined in [[5], pp.133].

### Condensed identity coefficients

While other programs are available for calculating condensed identity coefficients from pedigrees [6], we provide a function that is especially feasible for pedigrees with a large number of generations. Condensed identity coefficients can be derived from generalized kinship coefficients [7]. Bottom-up and top-down are two computational strategies for calculating generalized kinship coefficients from a pedigree. The bottom-up approach starts from the target individuals and moves up the pedigree until reaching the founders. It requires minimal storage but the computational load increases approximately exponentially with the number of generations. The bottom-up approach is computationally infeasible if both the number of generations and the number of individuals are large. The top-down approach starts from founders and moves down to the target individuals. The computational load is approximately linear in the number of generations. However, the intermediate generalized kinship coefficients need to be stored, which may require extensive storage if the number of individuals in a generation is large. We have implemented a function that allows users to adjust the arguments to achieve a hybrid bottom-up/top-down approach, which can accommodate very deep pedigrees.

### Variance components

QTLRel can estimate variance components given the appropriate incidence matrices. QTLRel estimates these variance components using maximum likelihood. These estimates are nearly equivalent to those obtained by restricted maximum likelihood for typical sample sizes. The maximum likelihood estimates are found numerically using one of several methods. We default to Nelder-Mead since we have found it to be more numerically stable. QTLRel allows users to select variance components using a model selection procedure or perform statistical significance tests for them.

### Genome scans

Re-estimating variance components at each marker in a genome scan may not be computationally feasible. The approach used by QTLRel is to first estimate the correlation matrix due to polygenic, residual and other random effects, which is based on the estimated variance components, and then use this matrix as known to scan the genome. Testing fixed effects conditional on estimated random effects is a general approach in mixed-effect models [2,3,8,9]. Our method is most similar to the measured-genotype fixed-heritability method in Aulchenko et al. [9].

### Empirical significance thresholds

QTLRel implements two methods for estimating genome-wide significance thresholds. The first is a permutation test in which the genotypes are permuted while the phenotypes and incidence matrices are held constant. We have previously demonstrated that when polygenic effects are ignored in the model type I error rates are inflated when a permutation is used; however, when the model is appropriate, permutation performs well [10]. The second method is gene dropping which can appropriately control type I error rates even when polygenic variation is ignored in the model.

## Results

QTLRel has been successfully used in an AIL to identify QTL for methamphetamine sensitivity [10], muscle weight [11], prepulse inhibition [12] and body weight [13]. In the analysis of body weight we calculated all five genetic incidence matrices, but only estimated additive and dominance variance components because the other variance components are negligible in general [14]. We then performed a genome scan using 688 individual genotyped at 3,105 SNP markers. We identified 11 QTL that exceeded the .05 genome-wide significance threshold estimated from 1800 gene dropping samples. These 11 QTL were confirmed as distinct signals using a forward step-wise multiple-QTL mapping function implemented in QTLRel. We also investigated genome-wide epistatic effects but found none. The analysis was accomplished on an Linux machine equipped with a 2.40GHz Intel(R) Core(TM)2 Quad CPU. It took 295 seconds to estimate variance components and 70 seconds to perform the genome scan.

## Conclusions

QTLRel provides a toolkit for genome-wide association studies that is capable of calculating genetic incidence matrices from pedigrees, estimating variance components, performing genome scans, and estimating significance thresholds. It can model interactive covariates and multiple genetic and non-genetic variance components. Other functions include multiple-QTL mapping and genome-wide epistasis. QTLRel can perform interval mapping based on the Haley-Knott method [15] for markers with 2 alleles. Because QTLRel is implemented in R users can take advantage of numerous other statistical packages; however, there is room to improve on QTLRel's speed since it makes use of many intermediate R functions. It is our intention to extend some functionalities, e.g., the Haley-Knott method to markers with more than two alleles. In summary, QTLRel provides a stand-alone, comprehensive tool to perform QTL analyses in populations in which relatedness is a concern.

## Availability

QTLRel is an R package. It is publicly available on R CRAN http://cran.r-project.org/web/packages/QTLRel for Windows, Linux and Mac machines under the GNU GPL license. A tutorial is available in the package as well as on the Palmer lab web page http://www.palmerlab.org/software.

## Authors' contributions

RC wrote the program, this paper and the tutorial and has made seminal intellectual contributions throughout this project. MA worked with the other authors and lent his considerable experience addressing similar issues in human populations such as the Hutterites. AAP initiated the projects that lead to the development of the software and has interacted extensively with all other authors. ADS worked with the other authors and lent his considerable experience with the analysis of animal breeding designs and helped to develop earlier versions of both this paper and the tutorial. All authors read and approved the final manuscript.
